# Cosmopolitan Distribution of *Endozoicomonas*-Like Organisms and Other Intracellular Microcolonies of Bacteria Causing Infection in Marine Mollusks

**DOI:** 10.3389/fmicb.2020.577481

**Published:** 2020-10-30

**Authors:** Irene Cano, David Ryder, Steve C. Webb, Brian J. Jones, Cara L. Brosnahan, Noelia Carrasco, Barbara Bodinier, Dolors Furones, Tobia Pretto, Francesca Carella, Bruno Chollet, Isabelle Arzul, Deborah Cheslett, Evelyn Collins, Karin B. Lohrmann, Ana L. Valdivia, Georgia Ward, María J. Carballal, Antonio Villalba, Ionan Marigómez, Stein Mortensen, Kevin Christison, Wakeman C. Kevin, Eduardo Bustos, Lyndsay Christie, Matthew Green, Stephen W. Feist

**Affiliations:** ^1^International Centre of Excellence for Aquatic Animal Health, Cefas Weymouth Laboratory, Weymouth, United Kingdom; ^2^Cawthron Institute, Nelson, New Zealand; ^3^Animal Health Laboratory, Ministry for Primary Industries, Upper Hutt, New Zealand; ^4^Institut de Recerca i Tecnologia Agroalimentaries (IRTA), Sant Carles de la Ràpita, Tarragona, Spain; ^5^Istituto Zooprofilattico Sperimentale delle Venezie, Legnaro, Italy; ^6^Department of Biology, University of Naples Federico II, Naples, Italy; ^7^SG2M-LGPMM, Laboratoire De Génétique Et Pathologie Des Mollusques Marins, Ifremer, La Tremblade, France; ^8^Marine Institute, Galway, Ireland; ^9^Departamento de Biología Marina, Facultad de Ciencias del Mar, Universidad Católica del Norte, Centro Innovación Acuícola Aquapacífico, Coquimbo, Chile; ^10^Life Sciences Department, Natural History Museum, London, United Kingdom; ^11^Centro de Investigacións Mariñas, Consellería do Mar da Xunta de Galicia, Vilanova de Arousa, Spain; ^12^Departamento de Ciencias de la Vida, Universidad de Alcalá, Alcalá de Henares, Spain; ^13^Research Centre for Experimental Marine Biology and Biotechnology (PIE), University of the Basque Country (UPV/EHU), Plentzia, Spain; ^14^Institute of Marine Research, Bergen, Norway; ^15^Department of Environment, Forestry and Fisheries, Cape Town, South Africa; ^16^Institute for International Collaboration, Hokkaido University, Sapporo, Japan; ^17^Centro Acuícola Pesquero de Investigación Aplicada (CAPIA), Universidad Santo Tomás, Sede Puerto Montt, Chile

**Keywords:** mollusk, OTU, Endozoicomonas, Chlamydiae, Mycoplasma, Francisella, Lucinoma, Ridgeia

## Abstract

Intracellular microcolonies of bacteria (IMC), in some cases developing large extracellular cysts (bacterial aggregates), infecting primarily gill and digestive gland, have been historically reported in a wide diversity of economically important mollusk species worldwide, sometimes associated with severe lesions and mass mortality events. As an effort to characterize those organisms, traditionally named as *Rickettsia or Chlamydia*-like organisms, 1950 specimens comprising 22 mollusk species were collected over 10 countries and after histology examination, a selection of 99 samples involving 20 species were subjected to 16S rRNA gene amplicon sequencing. Phylogenetic analysis showed *Endozoicomonadaceae* sequences in all the mollusk species analyzed. Geographical differences in the distribution of Operational Taxonomic Units (OTUs) and a particular OTU associated with pathology in king scallop (OTU_2) were observed. The presence of *Endozoicomonadaceae* sequences in the IMC was visually confirmed by *in situ* hybridization (ISH) in eight selected samples. Sequencing data also indicated other symbiotic bacteria. Subsequent phylogenetic analysis of those OTUs revealed a novel microbial diversity associated with molluskan IMC infection distributed among different taxa, including the phylum Spirochetes, the families *Anaplasmataceae* and *Simkaniaceae*, the genera *Mycoplasma* and *Francisella*, and sulfur-oxidizing endosymbionts. Sequences like *Francisella halioticida/philomiragia* and *Candidatus* Brownia rhizoecola were also obtained, however, in the absence of ISH studies, the association between those organisms and the IMCs were not confirmed. The sequences identified in this study will allow for further molecular characterization of the microbial community associated with IMC infection in marine mollusks and their correlation with severity of the lesions to clarify their role as endosymbionts, commensals or true pathogens.

## Introduction

The global production of marine bivalves is in a steady increase, with an estimated 15 million tonnes harvested per year (average data from 2010 to 2015) including both mariculture and fisheries (Wijsman et al., 2018). Following the trend from previous years, a global increase in demand and high prices were recorded in 2019 (FAO, 2020). National monitoring programs and health assessments are therefore conducted worldwide aiming to protect bivalve aquaculture and fisheries in natural beds. Since the 1970s, among the non-listed diseases by the World Organisation for Animal Health (OIE), numerous records of intracellular microcolonies (IMC) of bacteria have been reported worldwide, infecting economically important bivalve species (Harshbarger et al., 1977; Comps and Raimbault, 1978; Comps, 1980; Cano et al., 2018). Based on histological observations, those IMC of bacteria (intracellularly observed) or microbial aggregates (extracellularly) have been traditionally named as either *Rickettsia*-like organisms (RLOs) or *Chlamydia*-like organisms (CLO), however, those organisms remain largely uncharacterized.

Infections related to IMC in economically important marine molluskan species have been reported in Spain, infecting the common cockle *Cerastoderma edulis* (Carballal et al., 2001), the grooved razor shell *Solen marginatus*, the pod razor clam *Ensis siliqua* (Ruiz et al., 2013, 2015) and the Mediterranean mussel *Mytilus galloprovincialis* (Villalba et al., 1997); in France, infecting the Mediterranean mussel (Comps and Tigé, 1999), the European flat oyster *Ostrea edulis* (Comps et al., 1977; Comps, 1985b) and the wedge clam *Donax trunculus* (Comps, 1985a); in Chile, infecting the Chilean mussel *Mytilus chilensis* (Lohrmann et al., 2019) and the Peruvian scallop *Argopecten purpuratus* (Lohrmann et al., 2002; Lohrmann, 2009); in Norway, infecting the European flat oyster (Mortensen, 1993); in Italy, infecting the wedge clam (Carella et al., 2019); and in New Zealand, infecting the Pacific oyster *Crassostrea gigas* and the dredge oyster *Ostrea chilensis* (Hine, 1997, 2000; Diggles et al., 2002). In these reported cases, no host inflammatory response was observed and the severity of the lesions associated with the IMC infection was mild. In IMCs infecting mytilid species in Chile, a symbiotic relationship was then suggested (Lohrmann et al., 2019). On the other hand, IMC infections directly or indirectly associated with mortality have also been reported in the United States, infecting the sea scallop *Placopecten magellanicus* (Gulka et al., 1983; Walter et al., 2007), the Pacific razor clam *Siliqua patula* (Elston, 1986) and the abalone *Haliotis rufescens* (Moore et al., 2000); in France and the United Kingdom (UK) infecting the king scallop *Pecten maximus* (Le Gall et al., 1988; Cano et al., 2018); in the Philippines and the Federated States of Micronesia infecting the giant clam *Hippopus hippopus* (Norton et al., 1993); in China infecting the Suminoe oyster *Crassostrea ariakensis* and the blood clam *Tegillarca granosa* (Wu and Pan, 2000; Zhu et al., 2012); in Spain, infecting the banded carpet shell *Polititapes rhomboides* (Villalba et al., 1999); and in New Zealand infecting the toheroa *Paphies ventricosa* and the tuatua *Paphies subtriangulata* (Taylor, 2017a,b).

Infection and transmission are suspected to occur trough the water column. For the majority of the listed species above it is unclear which factors are determinants for the onset of disease. In the case of *rickettsia* infections in abalone *Haliotis* spp. it has been shown that temperature, host-susceptibility and pathogen-specificity can affect the growth of the IMCs and subsequently cause disease and mortality (Cruz-Flores and Cáceres-Martínez, 2020).

Intracellular microcolonies of bacteria appear mainly infecting either the digestive gland and/or gill epithelium, with sporadic observation in the adductor muscle, mantle, labial palps and intestine (Travers et al., 2015; Carballal et al., 2016; Webb and Duncan, 2019). *In situ* hybridization (ISH) showed that the IMC infecting king scallop was likely to be the same bacterium infecting different tissues throughout the animal (Cano et al., 2018). However, histopathology observations of several bivalve species reveal IMC infections of different sizes and shapes, supporting evidence for the presence of co-infections of more than one bacterium (Costa et al., 2012). IMCs infecting bivalves usually present as discrete basophilic inclusions of a range of sizes (colony areas ranging from 10^3^ to 10^5^ μm^2^) (Comps et al., 1980) mainly observed in the digestive gland and/or gill (Villalba et al., 1999; Cano et al., 2018). In some bivalve species, those inclusions can evolve to large extracellular cysts or xenoma, leading to tissue disruption mainly in the gill epithelium (Carballal et al., 2001) and subsequent release of bacteria into the interlamellar space with a potential shedding from host animals (Hooper et al., 2019). As a result of those reports, and the suspicion of mortalities associated with IMCs in some mollusk species, it is urgent to genetically characterize those IMCs to enable insight and control of the mollusk’s health. IMC infections related directly or indirectly to mass mortality in mollusks genetically identified so far include *Endozoicomonas*-like organisms (ELOs) infecting king scallop in the United Kingdom (Cano et al., 2018), deep-sea mussels from hydrothermal vents and cold seeps (Zielinski et al., 2009), Pacific razor clam (Elston, 1986; Kerk et al., 1992) and grooved carpet shell clams *Ruditapes decussatus* (Costa et al., 2012); and the rickettsia *Candidatus* Xenohaliotis californiensis, the causative agent of withering syndrome in farmed abalones (Moore et al., 2000). In those records of deep-sea mussels, mortalities were not clearly linked to parasitism as shifts in seepage also occurred (Ward et al., 2004; Mills et al., 2005).

However, the vast majority of IMC infecting mollusks remains unclassified, despite some of them being associated with population declines. This information is essential to establish the type of symbiosis they maintain with their host (mutualisms, commensalism or parasitism) and whether they can act as true pathogens and from there to determine the necessity of implementing control measures in aquaculture (Cruz-Flores and Cáceres-Martínez, 2020). In the present study, the microbial diversity associated to IMCs infecting economically important marine molluskan species was interrogated by a 16S rRNA gene amplicon sequencing and phylogenetic analysis approach, through international collaboration, to progress in the molecular identification of IMCs infecting mollusks. Differences in the severity of the pathology and their taxonomic classification are discussed.

## Materials and Methods

### Sample Collection

**FIGURE 1:**
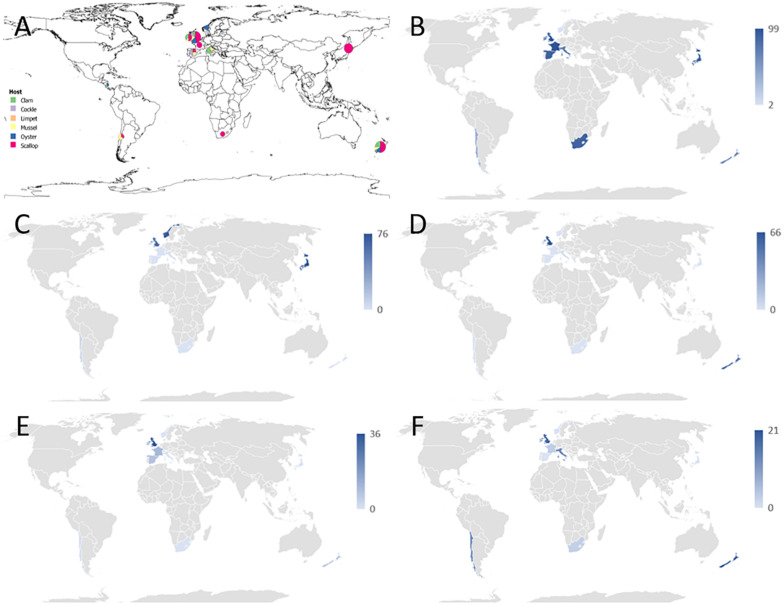
**(A)** Overview of the geographical distribution of mollusks collection received by the sequencing laboratory. For the simplicity of the drawing, mollusk species were identified as clams (colored in green), common cockles (in purple), limpets (in orange), mussels (in yellow), oysters (in blue), and scallops (in red). Each circle is scaled according to the number of samples per country. Samples from Nicaragua were not included in the 16S sequencing analysis. For the species details refer to Table 1. **(B–F)** Graphical distribution showing the maximum proportion of reads per country which align against Operational Taxonomic Units (OTUs) classified into a particular taxonomic category as follows: **(B)** *Endozoicomonas*-like organisms; **(C)** *Anaplasma* group. **(D)** *Francisella* group; **(E)** *Mycoplasma* group; **(F)** other symbionts, which included *Chlamydia*, Bacteroidetes, Spirochetes or sulfur-oxidizing endosymbiotic bacteria.

Marine mollusk collection was carried out as part of national surveillance programs from 2010 to 2018 (Figure 1A). Seventeen institutions from ten countries participated in this investigation. Twenty-two species were subjected to histological examinations in the laboratory of origin, comprising a total of 1950 specimens, after which a selection of 99 samples covering 20 species showing IMC of unknown etiology was sent to a single laboratory for 16S rRNA gene amplicon sequencing analysis (Table 1). Samples of Mangrove cupped oysters *Crassostrea rhizophorae* and slender marsh clams *Polymesoda arctata* (both sampled in Nicaragua), and king scallops (from Norway) were analyzed only by histology.

**TABLE 1:** Summary table of the molluskan samples analyzed, sequencing results and associated pathology.

Country	Host organisms	Common name	Year	16S amplicon sequencing					Histology IMC		
				Material	No samples	No OTUs	ID ELO OTUs	ID Other OTUs	Tissue	Prevalence	Pathology
Japan	*Mizuhopecten yessoensis*	Yesso scallop	2017	G-EtOH	7	1	6		G,D	100% of 7	N
				D-EtOH	7	2	6	56(A)			
New Zealand	*Pecten novaezelandiae*	New Zealand scallop	2017	G-DNA	7	2	3, 15		G	100% of 30	Y
			2015	G,M-DNA	2	3	3, 8, 15		G,GN	100% of 5	
				V-FFPE	1	6	1, 2, 3, 8, 15	10(A)			
	*Ostrea chilensis*	Dredge oyster	2014	G-EtOH	3	9	1, 2, 7, 8, 54, 99	25(A), 29(F) 155(M)	G,D	8.7% of 149	N
	*Paphies ventricosa*	Toheroa	2017	G,M-DNA	3	3	6	25(A), 165(E)	G,D,A,I	100% of 10	N
	*Panopea zelandica*	Geoduck clam	2014	G-EtOH	2	9	1, 2, 4, 7, 8, 54	10(A) 62, 165(E)	G	57% of 90	N
	*Perna canaliculus*	Green-lipped mussel	2014	G-EtOH	2	5	2, 4, 54, 99	155(M)	G	6% of 150	N
South Africa	*Pecten sulcicostatus*	Agulhas ridged scallop	2010/1	V-FFPE	4	3	1, 3	27(C)	G,D,M	62% of 45	N
United Kingdom	*Mytilus edulis*	Blue mussel	2013	D-DNA	4	7	1, 4, 8	10, 19, 30(A) 13(F)	D	7% of 30	Y*
	*Aequipecten opercularis*	Queen scallop	2017	G-EtOH	6	3	1, 3	27(C)	G	80% of 30	N
				D-EtOH	3	3	1, 3	14(M)			
	*Ostrea edulis*	Flat oyster	2016	D-EtOH	4	8	1, 2, 3 4, 7	51, 147(M) 53(C)	D	43% of 30	N
	*Cerastoderma edule*	Common cockle	2015	G-EtOH	2	1	7		G,D	100% of 30	N
	*Ruditapes philippinarum*	Manila clam	2017	V-EtOH	2	2	8	10(A)	G,D	50% of 30	N
	*Pecten maximus*	King scallop	2014	G-EtOH	1	1	2		G,D	100% of 30	Y
France	*P. maximus*	King scallop	2009 Pertuis Breton	D,G-EtOH	2	3	2	14(M), 27(C)	G,D	75.8% of 29	Y
			2009 Quiberon Bay	G-EtOH	3	3	2	14(M), 27(C)	G	73.3% of 15	
Ireland	*M. edulis*	Blue mussel	2016	G-EtOH	1	7	1, 3, 7, 8, 99	19(A), 165(E)	D	6.6% of 30	N*
	*R. philippinarum*	Manila clam	2014	D-EtOH	2	1	8		D	41% of 105	N*
				G-EtOH	1	6	1, 3, 7, 8, 15, 16		G		
	*P. maximus*	King scallop	2016	V-EtOH	4	6	1, 2, 4, 8	14(M), 27(C)	G	13.3% of 30	N
Italy	*Mytilus galloprovincialis*	Mediterranean mussel	2018	G-DNA	2	4	1, 6, 16, 99		G	6% of 30	N
	*Callista chione*	Smooth clam	2011	V-FFPE	3	8	1, 2, 3, 4, 8, 15	13(F), 697(B)	G,D	100% of 20	N*
	*Donax trunculus*	Wedge clam	2018	V-FFPE	2	7	1, 2, 3, 4, 7, 16	30(A)	G,D	20% of 30	N
	*Patella caerulea*	Mediterranean limpet	2018	V-FFPE	1	4	1, 2, 8	10(A)	D	4% of 10	N
Spain	*M. galloprovincialis*	Mediterranean mussel	2013	V-DNA	2	4	1, 4, 16, 99		G	13.3% of 15	N
	*C. edule*	Common cockle	2013	V-FFPE	1	4	1, 2, 3, 4		G	76% of 30	N*
			2014	V-DNA	2	1	1		G	July 40% of 30 Sep. 93% of 30	
	*Mimachlamys varia*	Variegated scallop	2012	V-DNA	2	5	2, 7, 16, 111	14(M)	D	16% of 30	N
	*Polititapes aureus*	Golden carpet clam	2010	V-FFPE	1	3	1, 2, 3		G,M	58% of 30	N
Norway	*P. maximus*	King scallop	2016/7	D-EtOH	0				No IMCs	0% of 90	N
	*O. edulis*	Flat oyster	2017	D-EtOH	4	3	2, 6	25(A)	D	18.7% of 32	N
Chile	*Mytilus chilensis*	Chilean mussel	2016	V-FFPE	4	8	1, 2, 4, 8, 99	14, 51(M) 53(C)	G,D	38% of 478	N
	*Argopecten purpuratus*	Peruvian scallop	2014	V-FFPE	2	6	1, 2, 4, 8	10(A), 13(F)	D	24% of 17	N
Nicaragua	*Crassostrea rhizophorae*	Mangrove cupped oyster	2012	V-FFPE	0				D	1.5% of 120	N
	*Polymesoda arctata*	Slender marsh clam	2013	V-FFPE	0				D	12% of 83	N
*Total number of samples analyzed:*				*Sequenced*	*99*				*Histology*	1950	

The identity number assigned to each Operational Taxonomic Unit (OTU) identified is listed under *Endozoicomonas*-like organisms (ELO) OTUs or listed as an OTU not related to *Endozoicomonas* (Other OTUs) as follows: A, *Anaplasma*-like; M, *Mycoplasma*-like; C, *Chlamydia*-like; F, *Francisella*-like; B, *Candidatus* Brownia rhizoecola; and E, endosymbionts related sequences. Material: type of sample provided from the laboratory of origin: ethanol fixed (EtOH), formalin-fixed paraffin-embedded tissues (FFPE), or DNA extracted in origin. No samples: number of samples per group that was subjected to 16S amplicon sequencing (total 99). No OTUs: quantity of OTUs found per group. Organs are identified as “G” gill; “D” digestive gland; “M” mantle; “GN” gonads, “A” adductor muscle, “I” intestine, and “V” various organs (for DNA extracted from FFPE). “Histology IMC” refers to the histological observation of intracellular microcolonies of bacteria (IMC) and/or bacterial aggregates. “Tissue” refers to the infected organs observed in the histology. “No IMCs” refers to the absence of IMC infection observed in the surveyed population. “Prevalence” refers to the percentage of specimens showing IMCs on the population sampled. Pathology refers to the observation of tissue inflammation or tissue disruption directly associated with the IMCs. (*) Refers to population declines observed during the sampling, however, a role of the IMC in the mortalities was not established at the moment of the sampling (either IMC not related with inflammation, other pathogens were identified and/or environmental causes were recorded during the mortalities).

### Histopathology

Sampled specimens were fixed for 24–48 h in either seawater Davidsons’s fixative (Shaw and Battle, 1957) or 10% neutral buffered formalin and stored afterward in 70% ethanol before processing using the standard histological protocol of each laboratory. Embedded paraffin blocks were then sectioned and stained with either hematoxylin and eosin, Giemsa and/or Feulgen following standard protocols (Howard et al., 2004).

In all the laboratories, a consensus IMC scoring system was adopted, which included: the number of specimens in each survey presenting IMCs (data expressed as IMC prevalence in the population); infected tissues; the intensity of the infection (noted as mild, moderate or severe) which was assessed by first performing counts of IMCs in a minimum of three fields of view for each affected tissue, at ×200 magnification. Mild infection was noted when IMC average <3, moderate infection for IMCs average between 3 to 10, and severe when the IMC average >10. Infection severity was also influenced by the growth/size of the bacterial aggregates which might impair normal tissue functioning and whether there was host response (inflammatory infiltrates) directly linked to the IMCs. Only the prevalence (not severity or intensity or host response) of infection was specifically noted in some samples collected in New Zealand and Japan. The gross pathology, population declines and environmental changes that might affect the population health were also noted.

### DNA Extractions

DNA was extracted from a total of 99 samples selected after histological examination either from ethanol fixed tissues or from formalin or seawater Davidson-fixed paraffin-embedded tissues (FFPE tissues) in the absence of ethanol fixed tissues. For a specimen of New Zealand scallop 2015, DNA was extracted in parallel from gill and mantle of ethanol fixed tissues and FFPE tissues (samples ID RA16079-1 (FFPEE) and W15-1131-1 (ethanol) in Supplementary 1) and both samples sequenced.

DNA extractions from ethanol fixed tissues were carried out in the laboratories of origin (indicated in Table 1 as “DNA extracted on origin”) or in the sequencing laboratory (indicated in Table 1 as “Ethanol fixed”). Extractions were prepared in sterilized cabinets. For tissues fixed in ethanol, a portion of the tissue was excised and rinsed in molecular grade water before the extraction. DNA was extracted either from the gill, digestive gland, or mantle. For a selection of animals, where IMCs were observed both in gills and digestive gland, DNA was extracted separately from both organs for sequencing purposes. At the sequencing laboratory, tissues were subjected to proteinase-K digestion and then DNA was extracted using an EZ1 DNA tissue kit in a BioRobot EZ1 (Qiagen) following the manufacturer’s instructions. When the DNA was extracted on origin, different extraction kits were used as follows: DNA was extracted from the gill and mantle of New Zealand scallop, collected in New Zealand, using the Qiagen QIAamp mini kit (Qiagen) as per the manufacturer’s protocol for the tissue using a total of 20 mg of tissue. DNA from gill of Mediterranean mussel and common cockle, both collected in Delta de l’Ebre (Spain), were extracted using the DNeasy Blood and Tissue Kit (Qiagen) following the manufacturer’s instructions. The DNA concentration was measured using a Nanodrop 2000c spectrophotometer (Thermo Fisher Scientific), and DNA quality was verified by electrophoresis on a 1% agarose gel stained with ethidium bromide. Blue mussels, sampled in the United Kingdom, were processed and DNA extracted as described elsewhere (Ward et al., 2016). In the absence of material preserved in ethanol, DNA was extracted from FFPE tissues at the sequencing laboratory from a pool of 5 10 μm-thick sections of embedded tissue, following the QIAamp DNA FFPE tissue kit (Qiagen) which involved optimized lysis conditions to minimize DNA cross-linking occurring during formalin or seawater Davidson fixation. The first cuts of the blocks were disposed to prevent contamination.

### 16S Amplicon Sequencing

Ninety-nine DNA samples were then subjected to 16S amplicon sequencing as described before (Pichler et al., 2018). Briefly, 5 ng of DNA was subjected to PCR to amplify a 400 bp section of the bacterial 16S rRNA gene spanning the V4 region, using 10 μM of tagged, indexed primers forward: 5′GTGCCAGCMGCCGCGGTAA3′ and reverse: 5′GGACTACHVGGGTWTCTAAT3′. Indexed primers were designed allowing the samples to be multiplexed. The high fidelity NEBNext PCR mix was then used with cycling conditions of 99°C for 30 s followed by 30 cycles of 98°C for 10 s, 55°C for 30 s, 72°C for 30 s and a final step of 72°C for 2 min. Each sample was subjected to triplicate PCR reactions to reduce the error rate. The PCR product was cleaned up using AMPure XP beads at a ratio of 0.8:1 to select the amplicons of the correct size (Bronner et al., 2014). The cleaned-up PCR product was eluted in 10 mM Tris pH 8.5. The quantity of tagged DNA was checked using the ProNex NGS Library Quantification Kit (Promega). The samples were diluted to 4 nM, pooled together, and denatured using 0.2 M NaOH. The pooled samples were finally diluted to 12 pM with HT1 buffer and run on an Illumina MiSeq using the 600 cycle V3 technology. To increase the diversity of the library, lumpfish (*Cyclopterus lumpus*) genomic DNA was added to the run.

### 16S Bioinformatics Analysis – Identifying Unique Sequence Variants

A total of 99 samples were analyzed across three separate runs using version 1.8.0 of the dada2 package (Callahan et al., 2016). The distribution of quality scores was visualized prior to trimming the raw sequencing data. The reads were trimmed to a length which approximately corresponded to the point at which the lower quartile fell below a value of ten when calculating parameters to describe the distribution of quality scores at each point in the sequencing cycle, following advice provided in the online version of the dada2 tutorial, available at the time of publication, which suggests that trimming the lowest quality sequencing data from the end of reads will improve the algorithm’s sensitivity to rare sequence variants. For the first run, the quality of the reverse reads was poor, and only the forward reads were used, which were trimmed 220 bp from the 5′ end. In the second sequencing run the forward and reverse reads were trimmed to 180 and 100 bp, respectively. In the third sequencing run the forward and reverse reads were trimmed to 250 and 100 bp, respectively. In addition to this, reads were excluded from further analysis if the number of errors in a read was estimated to be more than two in the second and third sequencing runs, with a slightly higher limit of four errors being permitted for the first sequencing run. The primers in this experiment were chosen such that the final amplicon size should be approximately 250 bp across most of the targeted species, thus the parameters used for trimming, as described above, resulted in the identification of sequences which mostly ranged in length between 220 and 250 bp, after processing of sequencing data. Following trimming of reads, error rate models were parameterized and reads were dereplicated, using default parameters, as described in the online tutorial. Unique sequence variants were identified using pooled data from across each sequencing run, except in the case of sequencing run two, where computational limitations lead to the ‘pseudo pooling’ approach being chosen instead. Chimeric sequences were identified and removed using dada2’s internal algorithm, which can identify chimeric sequences if they can be exactly reconstructed through combining a left and right segment from two more abundant ‘parent’ sequences. This approach partially relies on denoising of sequence data carried out by dada2 earlier in the analysis. Following this, the sequencing tables from each sequencing run were combined such that any identical sequences, excluding variation in sequencing length, were merged.

### Identifying the Intracellular Bacteria

A list was compiled of Operational Taxonomic Units (OTUs) which made up more than ten percent of reads in any one sample in the experiment and which was identified as being the most abundant sequence in at least one sample. These sequences were then aligned against the NCBI database using a nucleotide blast search. The threshold for filtering blast results was 80% identity. Any sequences which did not appear to belong to any taxonomic group or species known to exhibit symbiotic properties, after checking the top twenty highest scoring alignments, or which simply did not align well against any sequences present in the reference database, were excluded from further consideration. Following this, the process was repeated, in an iterative manner, in order to identify any sequences which could be associated with symbiosis, and which were still relatively abundant in the sample. All the OTU sequences discussed in this paper were found in abundances of at least 100 reads per a sample, in at least one of the samples. Sequences selected for further investigation were then subjected to phylogenetic analysis. Taxonomic groups used for phylogenetic studies included the genera *Endozoicomonas* (Bartz et al., 2018), *Anaplasma* and *Wolbachia* (Szokoli et al., 2016), *Mycoplasma* (Hicks et al., 2014), *Rhabdochlamydia* (Jouffroy et al., 2016) and *Francisella* (Keim et al., 2007). Sequences of interest were aligned, along with reference sequences, using version 4.7.04 of the MAFFT software (Katoh and Standley, 2013). Alignments were trimmed manually, using version 1.3.0 of the UGENE software (Okonechnikov et al., 2012), so as not to include any unknown or missing data at the 5′ or 3′ end of the alignment. The OTUs were then used to infer phylogeny, using version 1.6.10 of IQ-tree (Nguyen et al., 2015), which was parameterized to use the maximum likelihood general time-reversible model with equal substitution rates and base frequencies as well as four rate categories and allowing for a proportion of invariable sites. One thousand iterations of the Shimodaira–Hasegawa (SH)-like approximate likelihood ratio test (Guindon et al., 2010) were used for bootstrapping.

### Detection of ELOs by Specific PCR and Sanger-Sequencing and by *in situ* Hybridization

A specific PCR and nested PCR to detect ELOs were conducted on 63 samples collected during the surveys. Specific primers to amplify either a fragment of 407 bp of the king scallop-ELO 16S rRNA gene (IMC-F, IMC-R) (Cano et al., 2018) or a 1,408 bp fragment of the 16S rRNA gene using universal 16S rRNA primers (FD1, rP2) (Weisburg et al., 1991) were used.

Both strands of the PCR products were Sanger-sequenced using either the ABI Prism Dye Terminator cycle sequencing kit (PerkinElmer, United Kingdom) on an ABI 310 genetic analyzer at either Cefas (United Kingdom), the University of Naples Federico II (Italy) or the University of Valencia (Spain). The consensus sequence similarity was determined by BLASTn (NCBI nt database April 2020) (Altschul et al., 1990). A tree showing phylogenetic relationships between the PCR product sequenced, the king scallop-ELO 16S rRNA gene (KX780138) and other bacterial sequences were inferred using the maximum likelihood method in MEGA7 (Kumar et al., 2016).

For a selection of 8 samples where ELO sequences were inferred in the 16S amplicon analysis, sequential sections on silane-treated slides (Sigma-Aldrich) were subjected to *in situ* hybridization (ISH) using as a digoxigenin-labeled DNA probe a 527 bp fragment of the king scallop-ELO 16S rRNA gene (KX780138) by using the forward primer S-D-Bact-0008-a-S-20 and reverse primer S-^∗^-Univ-0536-a-A-18 (Suau et al., 1999) as described previously (Cano et al., 2018).

## Results

### Histological Observations Revealed the Worldwide Distribution of Intracellular Microcolonies of Bacteria Infecting Economically Important Marine Molluskan Species

**FIGURE 2:**
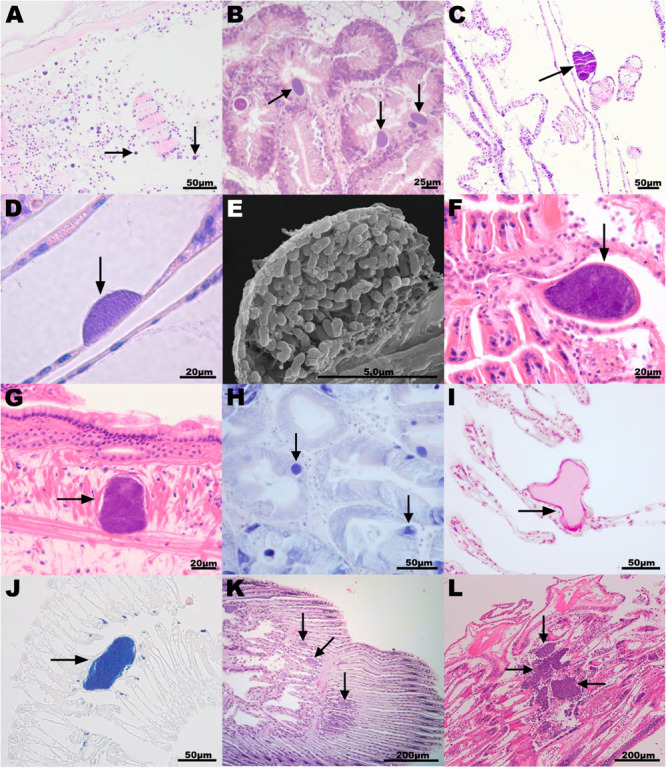
Histopathology associated with intracellular microcolonies of bacteria and bacterial aggregates infection in marine mollusks. **(A)** New Zealand scallop *Pecten novaezelandiae* gill section, origin New Zealand, hematoxylin and eosin (H&E) stain; **(B)** European flat oyster *Ostrea edulis* digestive gland section, Norway, H&E; **(C)** Agulhas ridged scallop *Pecten sulcicostatus* gill section, South Africa, H&E; **(D)** Chilean mussel *Mytilus chilensis* gill section, Chile, H&E; **(E)** Scanning electron microscope image of a Chilean mussel gill section showing detail of one bacterial inclusion; **(F,G)** Common cockle *Cerastoderma edule* gill **(F)** and mantle **(G)** section, Spain, H&E; **(H)** Wedge clam *Donax trunculus* digestive gland section, Italy, Giemsa stain; **(I,J)** Mediterranean mussel *Mytilus galloprovincialis* gill section, Italy, **(I)** Feulgen and **(J)** Giemsa stain; **(K)** Smooth clam *Callista chione* gill section, Italy, H&E; and **(L)** King scallop *Pecten maximus* gill, United Kingdom, H&E. Arrows point to the bacterial aggregates. H&E staining shows host cell nuclei (containing DNA) in blue and cytoplasm in pink. Bacterial aggregates appear stained in blue. Giemsa stain shows DNA in dark blue. Feulgen stain shows DNA in red.

National surveillance programs and historical data showed that IMC infecting marine molluskan species have worldwide distribution (Table 1 and Figure 2).

#### New Zealand

New Zealand scallops were sampled in 2015 (*n* = 5, prevalence and severity recorded) and 2017 (*n* = 30, only prevalence recorded) from a wild population from the Marlborough Sounds at the North of the South Island of New Zealand. No obvious external pathology was noted from the specimens; however, they were sampled from an area experiencing population declines. Histological examinations revealed organisms interpreted as IMC within the epithelial cells lining egg follicles, in the connective tissue subjacent to the labial palp epithelium, and in gills (Figure 2A). Inclusions that appeared as spherical microcolonies with densely packed basophilic bacteria-like organisms were interpreted as IMC. IMCs were observed in all animals analyzed in 2015 and 2017. For those sampled in 2015, severe damage and inflammation were observed in the organs where IMCs were present at high intensity in four of them and less so in one. Specimens of dredge oyster (*n* = 149), geoduck clam juveniles (*n* = 90), green-lipped mussel (*n* = 150) and toheroa (*n* = 10) were also analyzed. At the time of the sampling, no gross pathology was observed but after histopathological preparation, IMCs were mostly observed infecting gill or digestive gland. Historical records showed that only New Zealand scallops have been seen with extensive infections sometimes associated with distorted gill structures. Other hosts sampled in general had low intensity of infection with negligible apparent pathological effect.

#### Norway

A combination of farmed and wild populations of flat oysters and blue mussels are regularly sampled from 7 to 10 sites along the Norwegian coast, from the Swedish border and north to Trøndelag (report of the surveillance program in Mortensen et al., 2019). IMC infections have been observed in digestive gland and gill epithelium in both species, but usually at a low prevalence and never associated with pathology or mortality events. In a survey from Aga, Island of Bømlo consisting of 32 flat oysters sampled in 2017, the prevalence was 18.7% (Figure 2B). King scallops have been monitored irregularly since 1989, mainly as health monitoring of broodstock, but also as targeted surveillance of scallop broodstock in 2016–17 (*n* = 90). IMCs were not seen in the histology of the surveyed king scallop population, coinciding with historical records showing that IMC infections had never been observed in Norwegian scallops since surveys commenced.

#### South Africa

Specimens of agulhas ridged scallop were sampled as part of a health assessment for broodstock collection in False Bay 2010/2011. Mild infection of IMCs, observed as a low number of small basophilic bacterial aggregates, was observed in gill sections in 62% (28 out of 45) of the specimens sampled with no inflammation or gross pathology observed (Figure 2C).

#### Chile

The Peruvian scallop is cultivated mainly in Tongoy Bay northern Chile, and has been subjected to several surveys. The mean prevalences of IMCs detected were 39% from 305 scallops in Tongoy Bay (Lohrmann et al., 2002), 23% (range 0–57%) from 1080 scallops sampled in three northern bays seasonally (Lohrmann, 2009) and 24% in a sample of 17 juvenile scallops taken in austral winter 2014. From this last survey, two blocks were used for the molecular analysis. IMCs were only detected in the digestive gland, at low intensity of infection, with no histopathological damage. Chilean mussel cultivation is a very important activity in southern Chile. A health state assessment was undertaken on cultivated mussels from the three big culture zones and from two natural beds (where the seed for culture come from) in the Los Lagos Region. IMCs were observed mainly in cells of the branchial epithelium. The mean prevalence of 478 mussels was 38%, ranging from 0 to 60% with low intensity of infection and absence of inflammatory infiltrates (Figures 2D,E). IMCs were also detected occasionally in the digestive gland of mussels, with a prevalence of 0 to 1% (Lohrmann et al., 2019).

#### Spain

Golden carpet clams and common cockles were sampled in 2010 and 2013, respectively, from fished beds in Ría de Arousa (Galicia, NW Spain). None of the specimens analyzed showed gross pathology. However, histological examinations revealed large cysts surrounded by a fibrous eosinophilic cover and enclosing basophilic bacteria-like organisms in gills and rarely in mantle connective tissues. The number of these cysts was low in most cockles and clams and caused only local lesions (Figures 2F,G). The prevalence of these cysts was 58.3 and 76% for cockles and clams, respectively. IMCs (with a size of 20–60 μm) were also observed in the gills of common cockles collected between July (40% prevalence) and September 2014 (93% prevalence) in Alfacs Bay (Delta de l’Ebre, Catalonia, NE Spain). At the time of the collection, the common cockle population which was at a high density, was suffering mortalities of 80 and 40%, respectively, however, the role of the IMC infection in the population decline was not established due to the presence of other serious pathogens. IMCs were also observed in the gill tissues of Mediterranean mussels (13%) collected in the same location in January 2013, as well as in digestive gland of variegated scallops (16% prevalence) collected in March 2012 in Fangar Bay (Delta de l’Ebre, Catalonia, NE Spain).

#### Italy

In the Campania region (South of Italy), wild populations of limpets and wedge clams were sampled in spring 2018 and 2019 as part of surveillance programs. In the wedge clams, IMCs were present in the digestive gland and were small, pleomorphic, eosinophilic, and positive to Giemsa stain (Figure 2H). Bacterial infections did not cause significant histopathology but it was connected to infiltrative inflammations. The prevalence was 20% (6 out of 30) in 2018 and 16.6% (5 out of 30) in 2019. In limpets, the infection prevalence was 4% (2 out of 50) and not connected to any histopathological alteration. Samples of Mediterranean mussels were collected in April 2018 from local farms in Campania. IMC prevalence was 6% and not connected to any gross pathology. Gill IMC consisted of large rod-shaped, basophilic cells often observed to form large extracellular colonies. No inflammation was connected to the presence of IMC, which were positive to both Feulgen and Giemsa stain (Figures 2I,J). Smooth clams were sampled in 2011 during targeted surveillance of natural beds from an area experiencing population declines on the North-East coast of Italy (Figure 2K). The prevalence of IMC infection ranged between 80–100%, with most IMC observed on the gill epithelium and sporadically on the digestive gland.

#### Japan

Wild populations of yesso scallop were surveyed in Summer 2017 from Akkeshi Bay. Histological observations showed IMC infection in both gill and digestive tissues without apparent gross pathology. IMC prevalence was 100% (7 out of 7) of the specimens selected for histopathology assessment.

#### France

Wild adult king scallops were collected in the context of mortality events which occurred in Pertuis Breton and Quiberon Bay in May and July 2009, respectively. In scallops sampled in Pertuis Breton (Charente-Maritime), histological examination revealed the presence of IMC in epithelial cells of the digestive diverticula and gills in 22/29 individuals (light infection intensity). More precisely, 15/29 showed IMCs in gills only, 1/29 showed IMCs in gills and digestive diverticula, and 6/29 showed IMCs in digestive diverticula only. The presence of IMC was not systematically associated with haemocytic infiltration. In scallops from Quiberon Bay (Morbihan), histological examination revealed the presence of IMCs in epithelial cells of the gills in 11/15 individuals (7/15 light and 4/15 moderate infection intensity).

#### Ireland

Manila clams were collected between 2012 and 2014 as part of a study into ongoing mortality at a site in Drumcliffe Bay, Co., Sligo. Brown ring disease was evident in the samples collected and the presence of *Vibrio tapetis* was confirmed by PCR. IMC inclusions were observed in both high and low numbers in the digestive gland and gills in all years of the study. The mean prevalence was 41% in 105 Manila clams collected over the period. King scallops were collected in 2016 from an aquaculture site in Mulroy Bay during a routine health check. IMC inclusions were observed in low numbers in various organs in 4 out of 30 scallops with no associated mortality in the stock. The blue mussels were collected during a mortality event affecting mussels in Carlingford Lough in 2016. Environmental pressures were believed to have been at the root of the mortality, however, IMC inclusions were observed in high numbers in the digestive gland in 2 out of 30 mussels examined.

#### United Kingdom

Wild populations of king scallops, flat oysters, queen scallops, common cockles, and Manila clams were sampled from different locations on the South-West coast of England, while blue mussels were sampled on the Tamar estuary as part of routine surveillance programs. The prevalence of basophilic IMCs in the king scallop, queen scallop, and common cockle populations was 100%, with a higher number of IMCs observed on the gill epithelium (Figure 2L) and with fewer on the digestive gland. IMCs were occasionally seen, in low numbers (<5) in the adductor muscle and intestine. This finding was consistent with previous surveys (minimum of 30 specimens per species are collected every year). In the king scallop, IMC associated lesions were observed in the gills, with a severity ranging from moderate to severe. In recent years, the presence of severe IMC infection in gill tissues of king scallops has been associated with mass mortality events. In flat oysters, queen scallops, common cockles, and Manila clams the infection was mild (low number of IMCs per specimen and absence of inflammation) and there are no historical records of mortalities associated to IMC infection. In blue mussels and flat oysters, IMCs were more prevalent in the digestive gland. In the blue mussels population, the overall prevalence of IMCs was low (7%), however, in the infected animals the presence of IMCs was associated with host inflammation and the severity of the infection ranged from moderate to severe. The water quality was very poor (city discharges) when the blue mussels were taken.

#### Nicaragua

Mangrove cupped oysters and slender marsh clams were collected from a Nicaraguan Caribbean mangrove in 2012 and 2013, respectively. For both species, a low number of IMC inclusions corresponding to mild infections were observed in the digestive gland of the analyzed specimens, with no associated pathology. The estimated prevalence within each population was 1.5% for the mangrove cupped oysters (*n* = 120) and 20% (sampled in March, dry season, *n* = 10) and 11% (sampled in October, rainy season, *n* = 73) for the slender marsh clams. The low intensity and prevalence of the infection were similar to that reported in previous surveys conducted in the Caribbean coast of Nicaragua and Colombia (Aguirre-Rubi et al., 2018, 2019). Due to the low number of IMCs observed in positive samples, samples from Nicaragua were not included in the 16S sequencing study.

### Raw Sequencing Results

The median number of reads per sample was 130,103, before quality trimming and removal of chimeric sequences, and 41,698, after these steps had been carried out. The majority of tissue pieces were fixed using ethanol (*n* = 63) or had DNA extracted on-site (*n* = 17), though a small minority were fixed in formalin or seawater Davidson and subsequently embedded in paraffin (FFPE) (*n* = 19). Of those samples which fell below the lower quartile (9,752 reads per a sample), a majority corresponded with samples treated with formalin (*n* = 16), though samples treated or prepared in other ways were also represented within that group (*n* = 9). None of the samples treated with formalin had more than the median number of reads per sample (Table 2 and Supplementary 1).

**TABLE 2:** Summary table showing the minimum and maximum (Min_Max) number of total and filtered reads analyzed per group and the minimum and maximum proportion (%) of reads per group which align against OTUs classified into a particular taxonomic category.

Country	Host	Year	T	Fix.	N°	Total reads	Filtered reads	Minimum and Maximum proportion of reads						
								ELO	Anaplasma	Myco	Franc	Other	Envirom	Unclass
Japan	*Mizuhopecten yessoensis*	2017	DG	EtOH	7	1890_124065	184_22374	2_84	0_77	0	0	0	0.1_57	11_39
			G		7	190215_518086	42533_128106	53_89	0_2	0	0	0_0.004	0.06_0.3	10_46
New Zealand	*Pecten novaezelandiae*	2015	G, M	EtOH	2	201879_265388	152113_191950	33_39	0.007_0.009	0_0.006	0	0_0.002	2_21	45_57
			V	FFPE	1	15831	7721	9	0.2	0.05	0	0	31	59
		2017	G	EtOH	7	61504_497640	12554_108335	47_67	0_0.02	0	0	0_0.02	0.04_0.5	33_53
	*Ostrea chilensis*	2014	G	EtOH	2	46976_161146	36563_84196	4_35	0_0.3	0.05_0.3	0.2_56	0	2_7	36_57
	*Paphies ventricosa*	2017	G, M	EtOH	3	97852_402102	13639_69775	0.2_8	0.003_0.01	0	0	1_8	49_73	25_42
	*Panopea zelandica*	2014	G	EtOH	2	119124_200755	80651_128890	0_1	0_0.1	0	0	0.09_21	22_52	24_77
	*Perna canaliculus*	2014	G	EtOH	2	83306_309316	57205_145883	6_7	0	0.62_7.4	0.007_0.01	0	66_80	12_19
South Africa	*Pecten sulcicostatus*	2010/11	V	FFPE	4	213_6092	35_1027	63_88	0	0_0.19	0	0	6–17	4_17
United Kingdom	*Mytilus edulis*	2013	DG	EtOH	4	90113_340995	67417_259761	0.1_0.3	0_64	0	0.7_67	0	2_47	28_56
	*Aequipecten opercularis*	2017	G	EtOH	6	102788_1127022	78011_728073	68_98	0_0.008	0.008_0.4	0_0.005	0	0.3_14	1_16
			DG	EtOH	3	51328_595336	36838_448872	10_61	0_0.06	0.53_5	0	0_1	13_48	18_35
	*Ostrea edulis*	2016	DG	EtOH	4	67093_118337	880_81236	1_76	0.02_0.7	0_37	0_0.6	0_18	4_30	17_50
	*Cerastoderma edule*	2015	G	EtOH	2	256176_429593	198902_322093	67_81	0.01_0.02	0	0_0.002	0_1	0.8_0.9	17_31
	*Ruditapes philippinarum*	2017	V	EtOH	2	263257_462412	172345_280424	0.1_0.7	30_46	0_0.01	0_0.01	0	8_29	40_44
	*Pecten maximus*	2014	G	EtOH	1	197625	161027	99	0	0.02	0.006	0	0.1	0.2
France	*Pecten maximus*	2009	G, DG	EtOH	2	122866_534503	80998_418875	32_72	0	0.3_11	0_0.005	1_3	4_7	21_47
		2009	G	EtOH	3	214283_489903	168343_410408	84_100	0_0.01	0.03_0.4	0_0.01	0.001_0.3	0.07_1	0.1_13
Ireland	*Mytilus edulis*	2016	G	EtOH	1	417337	298547	0.2	0.009	0.003	0	0.01	91	7
	*Ruditapes philippinarum*	2014	DG	EtOH	2	279012_354851	221600_274155	55_61	0.11_0.15	0	0_0.01	0_0.3	0.3_0.4	38_44
			G	EtOH	1	25194	13342	2	0	0	0.02	0.01	68	29
	*Pecten maximus*	2016	V	EtOH	4	126068_572994	91340_417156	0.08_05	0	0.3_7	0_0.01	0.6_5	49_69	28_37
Italy	*Mytilus galloprovincialis*	2018	G	EtOH	2	20620_24086	3481_3820	59_70	0_0.08	0	0	0	0.6_1	28_40
	*Callista chione*	2011	V	FFPE	3	3992_26430	1606_11407	1_29	0_1	0	0_1.6	0_13	14_48	18_55
	*Donax trunculus*	2018	V	FFPE	2	8415_13030	864_4680	6_67	0_0.2	0	0	0	13_38	19_55
	*Patella caerulea*	2018	V	FFPE	1	15720	9858	2	0.3	0	0	0	19	78
Spain	*Mytilus galloprovincialis*	2013	V	EtOH	2	36506_190172	17380_142898	52_67	0_0.01	0	0_0.01	0	0.3_1	31_47
	*Cerastoderma edule*	2013	V	FFPE	1	24968	2670	28	0	0	0	0	14	57
		2014	V	EtOH	1	925235	768328	90	0	0	0.001	0	0.009	9
	*Mimachlamys varia*	2012	V	EtOH	2	83588_405889	56110_279185	0_17	0_0.05	5_8	0.003_0.06	0	13_16	60_78
	*Polititapes aureus*	2010	V	FFPE	1	2162	531	31	0	0	0	0	57	10
Norway	*Ostrea edulis*	2017	DG	EtOH	4	84350_268876	9646_41698	0.1_2	32_70	0	0	0_0.01	2_16	23_51
Chile	*Mytilus chilensis*	2016	V	FFPE	4	12087_64271	2631_28234	2_34	0_0.1	0_1.5	0_0.4	0_12	5_74	18_59
	*Argopecten purpuratus*	2014	V	FFPE	2	3306_12636	1083_1272	21_38	4_6	0_0.4	0_3	0	20_27	32_44

*ELO: Endozoicomonas-like organisms, Anaplasma group. Myco: Mycoplasma group. Franc: Francisella group. Other: shows the minimum and maximum proportion of reads with similarity to other symbionts, grouped either with Chlamydia, Bacteroidetes, Spirochetes or sulfur-oxidizing endosymbiotic bacteria. Envirom: environmental, shows the minimum and maximum proportion of reads that were not identified as symbionts and were then classified as environmental. Unclass: unclassified, shows the minimum and maximum proportion of reads per sample that were not included within the top ten percent of reads in any one sample or which were not identified as the most abundant in any one sample and which were therefore not investigated. T: tissue analyzed as follows: “G” gill; “D” digestive gland; “M” mantle, and “V” various organs (for DNA extracted from FFPE). Fix: DNA extracted from ethanol fixed (EtOH) or from formalin-fixed and paraffin-embedded (FFPE) tissues. N°: number of samples sequenced per group. Details of the metadata associated with each sample can be found in Supplementary Table 1.*

**FIGURE 3:**
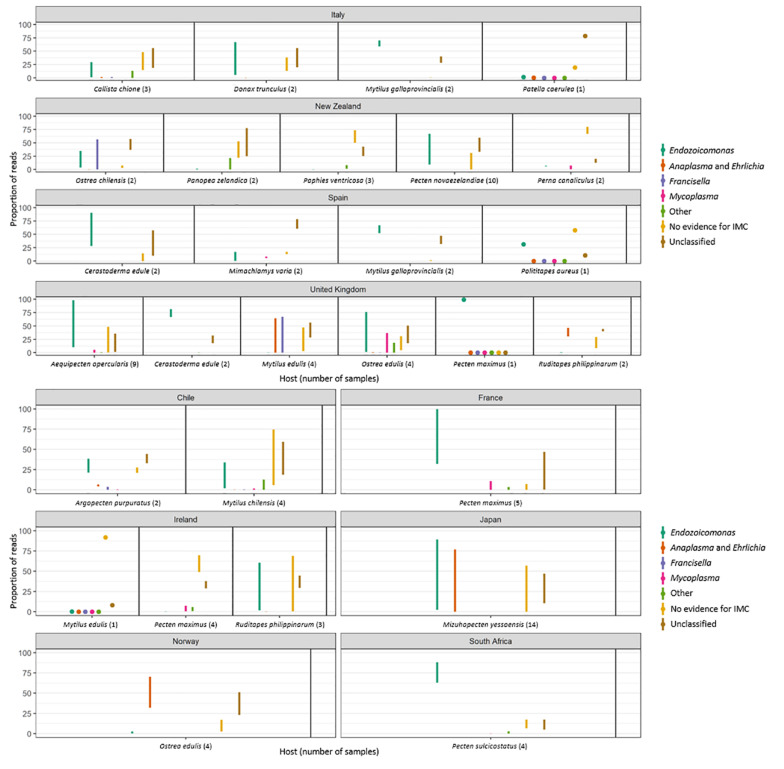
Plots showing the minimum and maximum proportion of reads per molluskan species and country which align against OTUs classified into a particular taxonomic category as follows: *Endozoicomonas*-like organisms; *Anaplasma* group. *Francisella* group; *Mycoplasma* group; other symbionts, which included reads mapping to *Chlamydia*, Bacteroidetes, Spirochetes or sulfur-oxidizing endosymbiotic bacteria. “No evidence for IMC” shows the minimum and maximum proportion of reads that were not identified as symbionts and were then classified as environmental. “Unclassified” shows the minimum and maximum proportion of reads that were not included within the top ten percent of reads in any one sample or which were not identified as the most abundant in any one sample and which were therefore not investigated.

**FIGURE 4:**
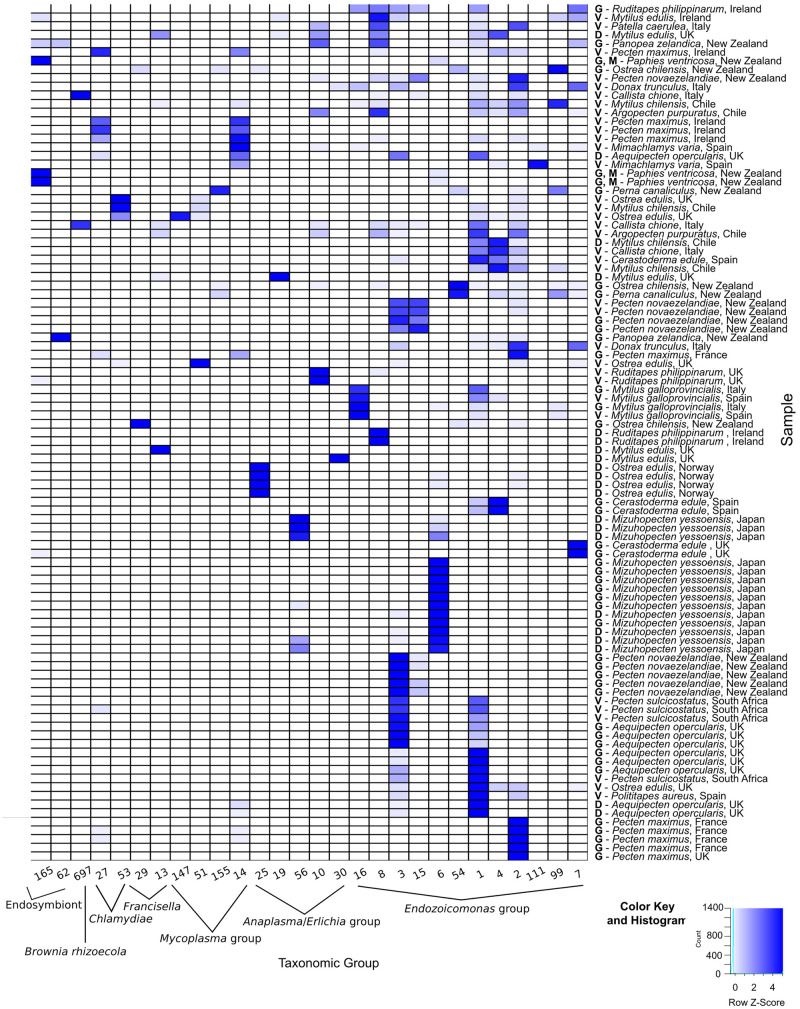
Heatmap of Operational Taxonomic Units (OTU) identified in molluskan samples. DNA for the 16S amplicon sequencing was extracted from either G = gill; D = digestive gland; M = mantle; GN = gonads; and V = various organs (for DNA extracted from formalin-fixed paraffin-embedded tissues). Numbers indicate the OTU sample identification (ID) shown on the phylogenetic trees in this figure.

The amplicon 16S rRNA gene sequencing identified a total of 28 OTUs related to intracellular parasites which were subsequently subjected to phylogenetic analysis. Of the 28 OTUs identified, 12 of them corresponded to family *Endozoicomonadaceae*, 5 to *Anaplasma* group, 4 to *Mycoplasma* group, 2 to *Francisella* group, 2 to *Chlamydia*, 2 were identified as endosymbionts, and 1 OTU corresponded to *Candidatus* Brownia rhizoecola (Figures 3, 4). None of the OTUs identified corresponded to the family *Rickettsiaceae*. Reads that were not identified as symbionts were classified as environmental, while a proportion of reads that were not included within the top ten percent in any one sample or which were not identified as the most abundant in any one sample were not investigated and assigned as unclassified.

The number of OTUs belonging to symbionts identified per sample ranged from 1 to 9. Despite the different average number of reads between FFPE and ethanol fixed, overall there were no significant differences in the final number of OTUs identified. For the New Zealand scallop 2015 specimen that was sequenced both from ethanol fixed (gill and mantle) and from FFPE (variety of tissues), three ELO OTUs identified in the ethanol sample were also seen in the FFPE sample, however, two other ELO OTUs and an OTU grouped with *Anaplasma* were also found in the FFPE sample (Table 1).

### Sequences Related to *Endozoicomonas*-Like Organisms (ELOs) Were the Most Prevalent Taxonomic Group Associated With IMC Infection in Marine Mollusks

The 16S amplicon sequencing revealed ELO-related sequences in all 20 molluskan species analyzed, however, geographical differences in the distribution of ELO sequences, were observed (Table 1 and Figure 1B). ELO sequences were identified in king scallops collected in the United Kingdom, France and Ireland, and European flat oysters collected in the United Kingdom. However, the same mollusk species sampled in Norway showed either the absence of IMC infections in king scallop or a low proportion of ELO sequences (minimum and maximum proportion of reads of 0 and 2%) in the case of European flat oysters, with the higher proportion of reads (32–70%) clustering within the genus *Anaplasma*. Similarly, Manila clams sampled in Ireland showed a high proportion of ELO related sequences (55–61%), while the same species sampled in the United Kingdom showed the higher proportion of reads related with *Anaplasma* (30–46%) and a low proportion of reads related with *Endozoicomonas* (0.1–0.7%).

New Zealand scallops, Mediterranean mussels, common cockle, and golden carpet clam showed ELO-related sequences independently of the organ analyzed and their geographical origin. All the seven samples analyzed from yesso scallop showed 100% of ELO related sequences in gill samples, however, digestive gland samples from the same animals revealed a mix of ELO with other taxa.

**FIGURE 5:**
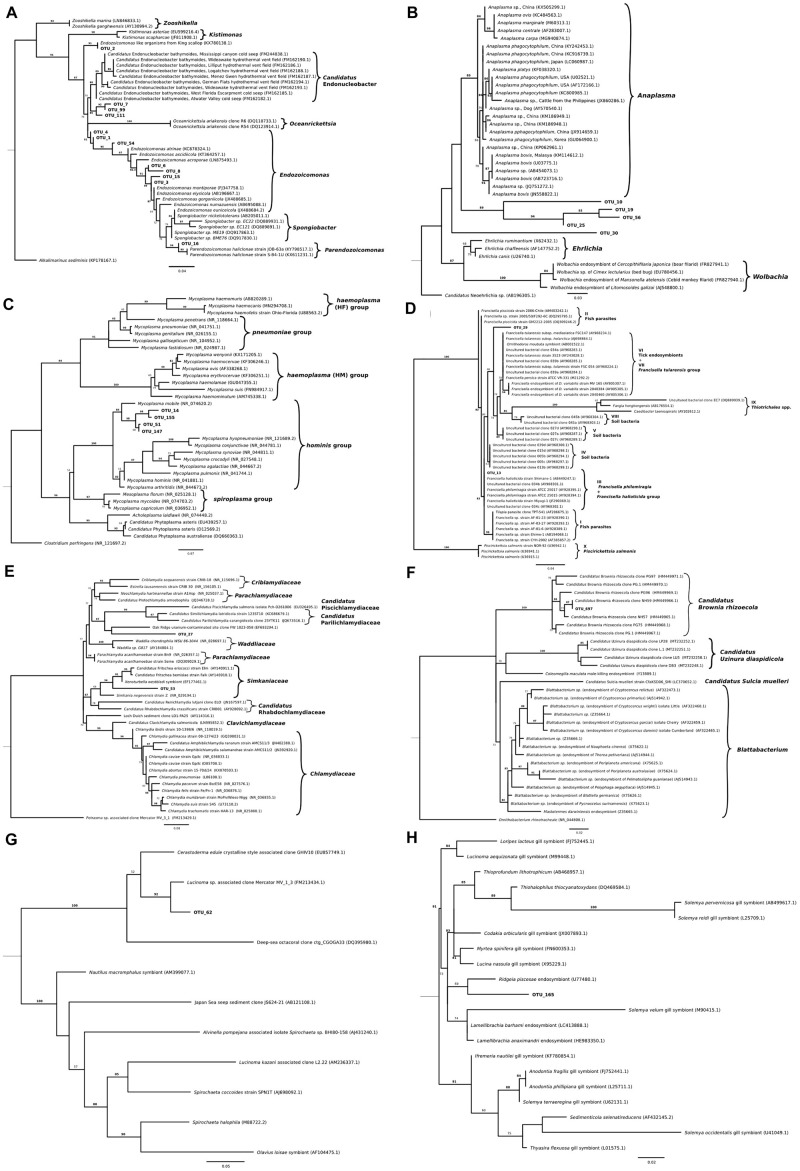
Maximum-likelihood trees showing phylogenetic relationships among partial sequences of the 16S rRNA gene of **(A)** *Endozoicomonas* (222 bp alignment), **(B)** *Anaplasma*, *Ehrlichia* and *Wolbachia* (221 bp alignment), **(C)** *Mycoplasma* (261 bp alignment), **(D)** *Francisella* (253 bp alignment), **(E)** *Chlamydia* (251 bp alignment), **(F)** Bacteroidetes (256 bp alignment), **(G)** Spirochetes (255 bp alignment), and **(H)** thioautotrophic gammaproteobacterium (223 bp alignment) related representatives as well as Operational Taxonomic Units (OTUs) which were found to closely align. For all the trees, IQ tree, version 1.6.10, was parameterized to use the maximum likelihood general time-reversible model with equal substitution rates and base frequencies as well as four rate categories and allowing for a proportion of invariable sites. A thousand iterations of the Shimodaira–Hasegawa (SH)-like approximate likelihood ratio test was used for bootstrapping. Values on each node represent the percentage of bootstrap replicates which support a given position when using the SH-like approximate likelihood ratio test. Branch lengths represent the number of substitutions per a base. For OTU distribution among molluskan species/country refer to Table 1.

Phylogenic analysis of partial sequences of the 16S rRNA gene of published *Endozoicomonas* and the identified ELO_OTUs infecting mollusks from this study showed sequences distributed along different lineages of *Endozoicomonadaceae* (Figure 5A). OTUs 2, 7, 99, and 111 were highly similar to the published genome of the ELO infecting king scallop and other *Endonucleobacter* sequences, with nucleotide identities ranging from 95 to 96% when aligned against *Ca*. Endonucleobacter bathymoides (FM162182.1). Interestingly, OTU 2 was the only ELO OTU identified in king scallops from the United Kingdom and France, those populations have been suffering declines. In those king scallops containing OTU 2 reads the presence of IMCs was linked to moderate and severe pathology.

Other *Endozoicomonas* OTUs not associated to pathology were identified, as OTU 16, showing a nucleotide identity of 98% with a strain of *Parendozoicomonas haliclonae* (NR_157681.1) and OTUs 1, 3, 4, 6, 8, 15, and 54 which clustered in the branch of *Endozoicomonas* isolates, showing nucleotide identities from 94 to 97% when aligned against *Endozoicomonas ascidiicola* (KT364260.1).

### 16S rRNA Gene-Based Analysis Showed a Novel Microbial Diversity Associated With Intracellular Microcolonies of Bacteria in Marine Mollusks

Reads not related to *Endozoicomonas* were discovered in 50% of blue mussels and 100% of Manila clams sampled from the United Kingdom, and in 100% of the European flat oysters analyzed from Norway. Phylogenetic analysis of OTUs showed relationships with taxa of intracellular parasites related to *Anaplasma* and *Francisella* in 50 and 20% of the species analyzed, respectively. Co-infections of ELOs with those and other intracellular bacteria belonging to *Mycoplasma* (observed in 35% of the mollusk species analyzed), *Chlamydiae* (25%), and a diverse group of endosymbionts (20%) were also identified but in lower proportion (Tables 1, 2 and Figures 1C–F).

#### *Anaplasma* Group

The most abundant bacteria taxon after *Endozoicomonas*-like organisms had sequences clustering with the genera *Anaplasma* (Figure 1C). Phylogenic analysis with the genus *Anaplasma* showed an independent cluster formed by the molluskan OTUs 10, 19, 25, and 56, with nucleotide identities ranging from 83.3 to 86.3% when aligned against a sequence from the *Anaplasma* clade (*A. phagocytophilum*, KC800985.1) (Figure 5B). OTU 30, present in blue mussel (United Kingdom) and wedge clam (Italy), was placed in an intermediate clade between *Anaplasma*, *Ehrlichia*, and *Wolbachia*, with nucleotide identity of 83.9% when aligned against a sequence from the *Ehrlichia* clade (*E.chaffeensis*, AF147752.2).

#### *Mycoplasma* Group

The third most abundant bacteria taxon contained 16S sequences related to *Mycoplasma* (Figure 5C). Bivalve IMC sequences from OTUs 14, 51, 147, and 155 clustered together with *Mycoplasma mobile* (NR_074620.2), showing nucleotide identities ranging from 90.4 to 92.9%.

#### *Francisella* Group

Two OTUs clustered with 16S sequences of the *Francisella* group. OTU 13 identified in blue mussel (United Kingdom), Peruvian scallop (Chile) and smooth clam (Italy) shared 100% nucleotide identity with *F. philomiragia* strain ATCC 25015 (AY928394.1) and *F. halioticida* strain Shimane-1 (AB449247). OTU 29 was exclusively identified in dredge oysters from New Zealand, with 95.2% of nucleotide identity with *Wolbachia persica* strain ATCC VR-331 (M21292.2) (Figure 5D).

#### *Chlamydia* Group

Two 16S sequences related to *Chlamydia*-like organisms were identified. The first one, OTU 53, infecting European flat oyster (United Kingdom) and Chilean mussel (Chile) hosts, showed a nucleotide identity of 86.9% when aligned with *Candidatus* Fritschea eriococci strain Elm (AY140911.1), and 87.7% with the chlamydiales symbiont of marine worms belonging to the genus *Xenoturbella westbladi*. The second one, OTU 27, showed a 79.6% nucleotide identity when aligned with the closest organisms, an uncultured bacterium from marine sediments (clone FW1023-058, EF693294) (Figure 5E).

#### Endosymbionts

Operational Taxonomic Unit 697 was found exclusively in 2 out of 3 smooth clams sampled in Italy and shared 100% identity with *Ca.* Brownia rhizoecola clone NH59 (HM449966.1) (Figure 5F) an insect endosymbiont described in mealybug. The DNA was extracted from FFPE tissues with an average number of reads after filtering of 1.1 × 10^4^. The average number of *Ca*. B. rhizoecola sequences in those samples was 370 (±233), while the presence of the most abundant *Endozoicomonas* OTU (OTU_1) was 100 (±67).

Two other OTUs related to endosymbionts were identified: OTUs 62 and 165. OTU 62, identified in geoduck clam from New Zealand, showed a 96% nucleotide identity with an endosymbiont of *Lucinoma* sp. (Bivalvia) clone Mercator MV_1_3 (FM213434.1) (Figure 5G). OTU 165, identified in the geoduck clam and toheroa from New Zealand, and in blue mussels from Ireland, showed a nucleotide identity of 94.5% with the closest relative, *Ridgeia piscesae* (tubeworm) endosymbiont (U77480.1) (Figure 5H).

### Detection of *Endozoicomonas* by Specific PCR and *in situ* Hybridization

The specific ELO-PCR was tested in a total of 63 samples containing 13 mollusk species (Table 3). The nucleotide sequence of the PCR products (∼407) of king scallop-collected in the United Kingdom and the OTU_2 identified in the same samples shared 100% homology with the king scallop-ELO (KX780138). The presence of ELO sequences was confirmed in a variety of samples (king scallop from France, wedge clam and smooth clam from Italy, common cockle from United Kingdom and Spain, Manila clam from Ireland, Mediterranean mussel from Italy, Chilean mussel and Peruvian scallop from Chile, and New Zealand scallop from New Zealand) showing nucleotide identities ranging from 92.9 to 100% when aligned against the king scallop-ELO (KX780138.1) (Supplementary 2, 3).

**TABLE 3:** Summary of samples subjected to PCR for the detection of the *Endozoicomonas*-like organism (ELO) 16S rRNA gene using the set of primers IMC-F, IMC-R (Cano et al., 2018), and the samples subjected to *in situ* hybridization (ELO ISH) using a 600 bp fragment of the king scallop-ELO 16S rRNA gene (KX780138).

Country	Host	ELO OTU	ELO PCR	ELO ISH
France	*P. maximus*	Y	+ (6/6)	
		Y	+ (3/3)	
Ireland	*P. maximus*	Y	+ (4/4)	
	*R. philippinarum*	Y	+ (1/1)	
United Kingdom	*P. maximus*	Y	+ (1/1)	G
	*M. edulis*	Y	− (4/4)	
	*C. edule*	Y	+ (4/4)	G
	*R. philippinarum*	N	− (2/2)	
New Zealand	*P. novaezelandiae*	Y	+ (3/3)	G
		Y	+ (2/2)	
	*O. chilensis*	Y	− (3/3)	
	*P. ventricosa*	Y	− (3/3)	
	*P. zelandica*	Y	− (2/3)	
	*P. canaliculus*	Y	+ (2/3)	
Italy	*M. galloprovincialis*	Y	+ (2/2)	
	*C. chione*	Y	+ (3/4)	G,D
	*D. trunculus*	Y	+ (3/5)	G,D
	*P. caerulea*	Y		D
Spain	*M. varia*	Y	− (2/2)	
	*C. edule*	Y	+ (2/2)	
Chile	*A. purpuratus*	Y	+ (2/2)	D
	*M. chilensis*	Y	+ (2/2)	G
Nicaragua	*C. rhizophorae*		− (2/2)	
	*P. arctata*		− (2/2)	

*ELO OTU column shows if ELO sequences were identified in the 16S amplicon sequencing analysis (marker as yes “Y” or no “N”). Samples from Nicaragua were not subjected to 16S amplicon sequencing. +: ELO-PCR positive, −: ELO-PCR negative. The number of specimens tested by PCR is shown in parenthesis (number of positive PCR/total samples analyzed by PCR). Positive ISH labeling was observed mainly in the gill “G” and/or in the digestive gland “D.” Blank means not tested.*

The Sanger sequencing of the ELO-PCR product obtained from variegated scallops (Spain) and geoduck clams (New Zealand) showed mix chromatograms indicative of the presence of more than one ELOs and/or another(s) bacterium(a) genome. Similarly, the sequencing of PCR products obtained with the set of universal primers (FD1, rP2, band of ∼1,408 bp) also showed a mix of sequences. In those populations, several ELO_OTUs and OTUs from other taxa were identified in the 16S amplicon sequencing analysis.

The ELO-PCR was negative in United Kingdom blue mussels and Manila clams. In those populations the proportion of ELO_OTUs identified was very low (<0.7%). The ELO-PCR was also negative for New Zealand dredge oysters and toheroas despite of the proportion of ELO_OTUs identified were higher (maximum proportion of ELO reads of 35 and 8%, respectively). Negative ELO-PCR was also obtained from the Nicaraguan mangrove cupped oysters and slender marsh clams. Those populations showed a very low intensity of IMC infection.

**FIGURE 6:**
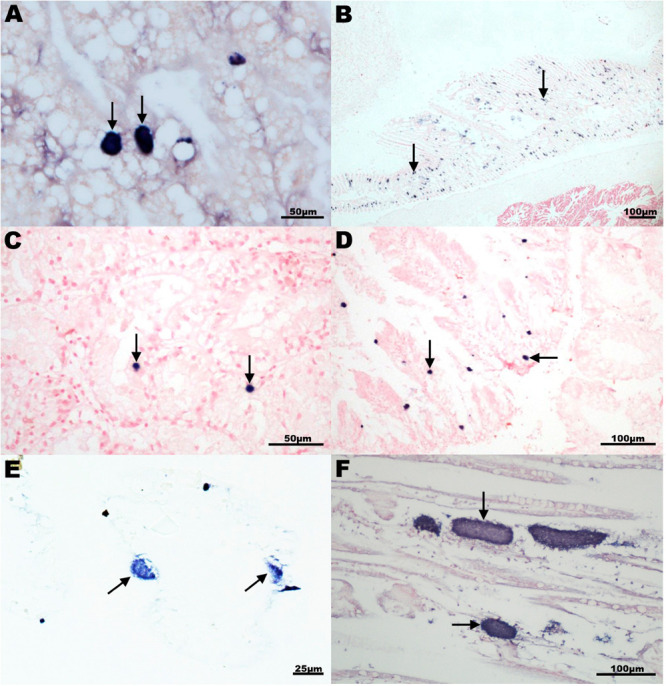
*In situ* hybridization analysis of intracellular microcolonies of bacteria (IMC) and extracellular bacterial aggregates in mollusks using a 16S rRNA gene probe generated with a 527 bp fragment of the king scallop *Endozoicomonas*-like organism (KX780138). Specific dark blue labeling (arrows) observed either in digestive gland **(A,C,D)** or gill section **(B,E,F)** of **(A)** Peruvian scallop *Argopecten purpuratus*, Chile; **(B)** common cockle *Cerastoderma edulis*, United Kingdom; **(C)** smooth clam *Callista chione*, Italy; **(D)** Mediterranean limpet *Patella caerulea*, Italy; **(E)** Chilean mussel *Mytilus chilensis* Chile; and **(F)** king scallop *Pecten maximus*, United Kingdom.

*In situ* hybridization was conducted on a selection of 8 species using a probe targeting the king scallop-ELO (Table 3). Labeling of bacterial colonies was observed in the gill epithelium of New Zealand scallop (New Zealand), Chilean mussel and Peruvian scallop (Chile), common cockle (United Kingdom) and smooth clam (Italy); and in the digestive gland of smooth clam, Mediterranean limpet and wedge clam (Italy) (Figure 6). In those species, OTUs related to the *Endozoicomonas* group were also identified.

## Discussion

An international collaboration has allowed the analysis of the microbial community associated with intracellular microcolonies of bacteria infecting economically important marine molluskan species. The 16S rRNA gene phylogeny performed indicated that *Endozoicomonadaceae* were the most abundant bacteria family infecting marine mollusks. A recent taxonomic revision has suggested the inclusion of the family *Endozoicomonadaceae* to a new family *Zooshikellaceae* (Liao et al., 2020). This multi-host bacterial family can, therefore, infect different species of bivalves such as oysters, cockle, clams, mussels, and scallops, as well as the marine gastropod limpet, occurring in geographically distant countries from Europe, Australia, Asia, Africa, and South America.

The mollusks were harvested either from managed fisheries located in natural beds or culture systems in open waters. Given the historical records of IMCs infecting bivalves, it seems unlikely that the wide geographical distribution of Endozoicomonadaceae in bivalves could be attributed solely to the international commercial trade of infected seedstock rather than the natural distribution, adaptation, and evolution of this bacterial group.

Phylogenic analysis of Endozoicomonadaceae OTUs placed the identified sequences within the sublineages *Endonucleobacter*, *Endozoicomonas*, and *Parendozoicomonas*. However, sequences related to severe lesions were grouped within the *Endonucleobacter* clade. In this group, OTU 2 was the only ELO OTU found in samples from king scallops showing moderate or severe lesions in the gill epithelia, collected from natural populations of the United Kingdom and France coasts. In the United Kingdom and France, king scallop populations have been historically associated with mass mortality events (Le Gall et al., 1988; Cano et al., 2018). This group also comprised the 16S sequence of the intranuclear pathogen, nuclear inclusion X (NIX), associated with massive mortalities of the Pacific razor clam in the United States (Elston, 1986; Kerk et al., 1992) and sequences from *Endonucleobacter bathymodioli* strains. Other OTUs within this clade were identified in toheroa sampled in New Zealand associated recently with population decline and events of mass mortalities (Taylor, 2017a). OTU 1 showed an intermediate position within *Endonucleobacter* and *Endozicomonas* clades and was present in 62% of the bivalve species analyzed, associated with mild to moderate lesions. Other Endozoicomonadaceae OTUs were identified in samples where the presence of IMCs was not associated with pathology (i.e., samples from scallops collected in South Africa), which might suggest differences in pathology associated with different *Endozoicomonas* species. Differences in host susceptibility and environmental factors might also contribute to the severity of the lesions. The identification of factors favoring the onset of the disease requires further investigation.

For those hosts where different infected organs from the same specimen were analyzed (gill and digestive gland from yesso scallop from Japan, queen scallop from the United Kingdom and Manila clam for Ireland), the same ELO_OTU was identified across the different infected organs (OTU 6, 1, and 8 for each specimen, respectively) suggesting a systemic infection throughout the animal. This result confirms previous work showing that the ELO infecting king scallop was likely to be the same organism infecting gills and the intestinal epithelium (Cano et al., 2018). However, in other samples, several Endozoicomonadaceae OTUs were identified infecting the same host, suggesting either the presence of different species and/or an incomplete identification at the species level. For some specimens where material preserved in ethanol was not available, DNA extracted from FFPE tissues was included in the 16S sequencing. The average number of reads obtained from FFPE tissues was lower than the reads obtained from ethanol-fixed tissues, nevertheless, Endozoicomonadaceae OTUs were also identified in those samples of inferior quality, indicating a high abundancy of Endozoicomonadaceae sequences in the analyzed samples. A particular specimen that was sequenced both from ethanol and FFPE showed a higher number of OTUs in the FFPE sample which could be attributed to an incomplete identification due to poor quality of the DNA after the formalin fixation. The length of fixation in formalin has been shown to affect the success of bacterial identification by 16S sequencing due to cross-linking of histone-like proteins to DNA or fragmentation of genomic DNA (Imrit et al., 2006). The DNA extracted from FFPE tissues and sequenced also included a variety of organs present in the histology block, while most of the DNA extracted from ethanol samples included a single tissue or a pool of two tissues containing the IMCs.

The second most prevalent bacterial taxon found in mollusks corresponded to *Anaplasma* related sequences. Five *Anaplasma*-related OTUs (OTUs 10, 19, 25, 30, and 56) were identified infecting 50% (10 out of 20) of the species analyzed. The *Anaplasma* related OTU 25 was highly prevalent (32–70% reads) identified in European flat oysters from Norway. Historically, IMC infection within the Norwegian population has not been associated with pathology or mortality events. Ehrlichiosis and anaplasmosis are rickettsial diseases in humans transmitted by tick vectors and caused by obligate intracellular bacteria in the genera *Ehrlichia*, *Anaplasma*, and *Neoehrlichia* (Bakken and Stephen Dumler, 2015). Given the low nucleotide identity of the OTUs with sequences of *Ehrlichia* and *Anaplasma* (∼84%) and that the phylogenetic analyses showed a clearly distinct and independent clade formed by the molluskan OTUs, our analysis suggests the discovery of a novel group of intracellular organisms. A follow-up study, using shotgun metagenomics, will allow for a comprehensive taxonomic classification and characterization of those novel organisms (Franzosa et al., 2015).

*Mycoplasma* related sequences were identified across 35% (7 out of 20) of the molluskan species analyzed, including scallops, oysters, and mussels. Mycoplasmas generally have fewer than 1000 genes, perform little *de novo* synthesis of required precursors, and are obligate commensals or parasites (Dandekar et al., 2002). OTUs related to the molluskan intracellular bacteria clustered specifically with *M. mobile*, showing nucleotide similarities >90%. A pathogenic *M. mobile* was originally isolated from the gills of a freshwater fish, the tench *Tinca tinca* L, showing a characteristic fast gliding motion (Kirchhoff and Rosengarten, 1984). Other mycoplasmas have been reported historically from fish-derived cell lines (Emerson et al., 1979), and fish tissues, in particular, *Acholeplasma laidlawii* considered to be a non-pathogenic microorganism (McMurtrie et al., 2019). In the present study, all the samples with mycoplasma sequences showed co-infections with Endozoicomonadaceae. Further work is therefore required to understand the role of *M. mobile*-like organisms in the development and severity of the lesions.

Within the *Francisella* group, OTU 13 was identified in 15% (3 out of 20) of the bivalve species analyzed: blue mussels (United Kingdom), Peruvian scallops (Chile), and smooth clams (Italy). In the absence of Endozoicomonadaceae sequences, the development of IMC cysts in the digestive gland of blue mussels was exclusively associated with *Francisella* and *Anaplasma*-like organisms. OTU 13 showed an identical nucleotide sequence (100%) with both *F. philomiragia* and *F. halioticida*. *F. philomiragia* is a human pathogen that can induce granulomatous disease (Sicherer et al., 1997). It has been isolated from muskrat *Ondatra zibethica*, water and other environmental samples (Jensen et al., 1969). The second species, *F. halioticida,* was first reported associated with a mass mortality event of the giant abalone *Haliotis gigantea* in Japan. The bacterium was isolated from infected hemolymph and its pathogenicity confirmed by experimental infections, reaching cumulative mortality of 98.6% (Kamaishi et al., 2010). Further taxonomic studies classified *F. halioticida* strain Shimane-1 as a novel organism, sharing a nucleotide identity of ∼98% of the 16S rRNA gene with isolates of *F. philomiragia* and *F. noatunensis* (Brevik et al., 2011). Later on, *F. halioticida* was recorded causing mortality in yesso scallops in Canada (Meyer et al., 2017) and Japan (Kawahara et al., 2018, 2019), and more recently reported in mussels *Mytilus* spp. experiencing mortalities in France (Charles et al., 2020). In those reports, *F. halioticida* occurred mostly intracellularly but without forming microcolonies similar to those typical of IMC; the infection was associated with severe inflammatory reactions, frequently associated with the formation of granuloma-like structures in the infected yesso scallops (Kamaishi et al., 2010; Meyer et al., 2017; Kawahara et al., 2018) and in *Mytilus* spp. (Charles et al., 2020). In the present study, due to the short length of the sequence, it was not possible to discriminate whether the organisms found in blue mussels, Peruvian scallops and smooth clams were *F. halioticida* or *F. philomiragia*. Blue mussels sampled in the Tamar estuary (United Kingdom) were exposed to city discharge waters. Whether the blue mussel *F. philomiragia*/*F. halioticida*-like organism is a true bivalve pathogen (similar to *F. halioticida*) or if its detection was due to environmental bioaccumulation from contaminated waters (as shown for *F. philomiragia*) requires further research, especially due to the risk of zoonotic disease.

Another *F. philomiragia*-like organism, sharing 98.6% nucleotide identity with OTU 13, has been reported from visceral granulomas in Taiwanese and Costa Rican tilapia *Oreochromis* sp. farms, associated with systemic IMC infections (Hsieh et al., 2006; Soto et al., 2009). The second OTU within the *Francisella* group, OTU 29, was identified in dredge oysters. Due to the low nucleotide identity with members of the group (95%) this organism will likely constitute a novel species of the family *Francisellaceae*.

Sequences within the Chlamydiae phylum were obtained in 25% (5 out of 20) of the mollusk species analyzed, grouped in OTUs 53 and 27. OTU 53, displayed the closest nucleotide similarities (∼87%) with *Ca.* F. eriococci and a symbiont of *Xenoturbella westbladi*, followed by 85.3% identity with *Simkania negevensis*, all of them belonging to the family *Simkaniaceae*. Sequences of *S. negevensis* have been identified in IMC infecting pearl oysters *Pinctada maxima* in Western Australia during an outbreak investigation into Oyster Oedema Disease (OOD). Although a link of IMCs with OOD-affected pearl oysters was suspected, the actual role of *S. negevensis* in the outbreak remains inconclusive (Crockford and Jones, 2012). *Ca.* F. eriococci has been found in the gut of whitefly *Bemisia tabaci* and scale insect *Eriococcus spurius* (Everett et al., 2005), while *X. westbladi* infects gastrodermal cells of benthic worm species of the genus *Xenoturbella*, presenting a limited host cell response not associated with cytopathological effects (Israelsson, 2007). OTU 27 was identified exclusively in agulhas ridged scallops. The closest organism to OTU 27 (79.6% nucleotide identity) was an uncultured bacterium associated with bioremediation of metals in contaminated sediments (Cardenas et al., 2008). Both OTUs 53 and 27 likely will constitute novel species within the Chlamydiae phylum.

Among the sequences related to endosymbionts, OTU 697 was identified as a clone of *Ca.* B. rhizoecola NH59, an endosymbiont bacterium of the plant-parasitic insect mealybug (Gruwell et al., 2010), within the phylum Bacteroidetes, family *Blattabacteriaceae*. There are no previous records of *Ca*. B. rhizoecola strains from marine hosts. The presence of *Ca*. B. rhizoecola DNA in the IMC of the smooth clams was not confirmed by ISH, thus this finding requires further investigation to rule out environmental contamination.

Another endosymbiont, OTU 62, found in geoduck clams from New Zealand, was located within the Spirochetes phylum, and clustered with an endosymbiont identified from the gills of the northern lucina clam *Lucinoma* sp., sampled from mud volcanoes of the Gulf of Cadiz (South Spain) (Rodrigues et al., 2010). Records of another spirochete species identified in a Nile deep-sea species of a cold lucina clam, *L. kazani*, showed a symbiotic relationship of the autotrophic sulfur-oxidizing bacteria with its host (Duperron et al., 2007). Other genetically distinct spirochetes have also been described infecting common cockles and the Pacific oysters *Crassostrea gigas*, although the role as endosymbiont or potential pathogen was not clarified (Husmann et al., 2010).

Operational Taxonomic Unit 165, identified in the geoduck clam and toheroa from New Zealand, clustered with sulfur-oxidizing endosymbiotic bacteria, with the closest relative being an endosymbiont from *R. piscesae*, a polychete annelid worm from deep-sea hydrothermal vents and cold-water seeps (Feldman et al., 1997). Those polychete worms rely on their sulfur-oxidizing endosymbionts for nutrients.

In summary, the bacterial community associated with IMC infection has been analyzed in economically or culturally (as for toheroa) important marine mollusk species worldwide. Phylogenetic analysis identified Endozoicomonadaceae sequences as the most abundant bacterial group associated with IMCs, showing a cosmopolitan distribution and adaptation of this bacteria group among molluskan species. Other symbiotic bacterial groups, belonging to *Anaplasma*, *Mycoplasma*, *Francisella*, *Chlamydia*, Bacteroidetes, Spirochetes, and sulfur-oxidizing symbionts were identified in some populations, in particular, *F. philomiragia*/*F. halioticida*, and B. rhizoecola like organisms.

The present study has several limitations. Although the 16S amplicon sequencing allowed an affordable sequencing of a large number of samples, it might present a limited taxonomical resolution (Bukin et al., 2019). PCR amplification might differ between templates due to the primers affinity. Inconsistencies in the taxonomic resolution across taxa might also affect the interpretation of the data (Soergel et al., 2012). Longer reads obtained by nanopore sequencing (Gonçalves et al., 2020) and better reference genomes might aid the identification to the species level. Although this study has revealed a preliminary identification of the bacterial community present in molluskan showing IMC, more analyses are required to unequivocally associate those sequences with the lesions and to establish relative abundances across these bacterial populations. Further studies encompassing ISH studies, laser capture of IMC lesions and sequencing, bacterial DNA enrichment protocols, and *in vitro* cultivation might offer a better characterization of those organisms and their role on the IMC and associated pathology. Traditionally, the *in vitro* isolation and culture of those organisms has been challenging, thus preventing fulfilment of Henle-Koch postulates. Nevertheless, the OTUs identified in this study will be used to design diagnostic PCRs and DNA probes for national surveillance programs which will allow for the implementation of measures to avoid the spread of potentially more virulent strains of those bacterial species associated with mortality events or population decline.

## Data Availability Statement

The datasets presented in this study can be found in online repositories. The names of the repository/repositories and accession number(s) can be found in the article/Supplementary Material.

## Author Contributions

SW, BJ, CB, NC, BB, DF, TP, FC, BC, IA, DC, EC, KL, GW, MC, IM, SM, KC, WK, EB, and AV contributed to resources. LC, IC, MG, and SF contributed to investigation. IC and DR contributed to formal analysis. IC and LC contributed to methodology. IC, KL, ALV, AV, SW, and DR contributed to original draft preparation. SF contributed to funding acquisition. All authors contributed to manuscript corrections and have read and agreed to the published version of the manuscript.

## Conflict of Interest

The authors declare that the research was conducted in the absence of any commercial or financial relationships that could be construed as a potential conflict of interest.

## Supplementary Material

Supplementary 1 Table summarizing the metadata associated with each sample (ID, host, fixation, date, tissue sequenced), the number of reads per sample obtained in the 16S rRNA gene amplicon sequencing (before and after filtering) and the number of reads per taxonomic group. The environmental column shows the number of reads per sample that was not included in the phylogeny study as there were not identified to belong to any endosymbiotic bacteria. The unclassified column shows the number of reads per sample that were not included within the top ten percent of reads in any one sample or which were not identified as the most abundant in any one sample and which were therefore not investigated through detailed phylogenetic analysis or alignment using BLAST.

Supplementary 2 Specific PCR for the detection of *Endozoicomonas*-like organisms (ELO) using the set of primers IMC-F and IMC-R described in Cano et al. (2018). Agarose gels showing a fragment of either 407 bp (first round of PCR, panel **B**) or 282 bp (second round of PCR, panels **A,C**) of the 16S rRNA gene. **(A)** Wedge clam *Donax trunculus* (samples ID from 1 to 5) and smooth clam *Callista chione* (samples ID from 6 to 9) collected in Italy. **(B)** King scallop *Pecten maximus* collected in France (samples ID 9 to 15). **(C)** Common cockle *Cerastoderma edule* collected in the United Kingdom (samples ID 16 to 19). DNA was extracted either from formalin-fixed paraffin-embedded tissues in duplicate **(A)** or from ethanol fixed tissues **(B,C)**. Positive DNA of king scallop *Pecten maximus* gill tissue was used as positive control (+ve). Water was used as negative control (−ve). 650 ng of a 100 bp DNA ladder (Promega) was used as molecular weight marker (M).

Supplementary 3 Maximum-likelihood tree showing phylogenetic relationships among the 16S rRNA gene of PCR sequenced products and a selection of *Endozoicomonas*-like organisms (ELO) and other symbiotic bacteria. Sequence name shows the name of the host, sample ID, country of origin, and tissue (G: gill, DG: digestive gland, V: variety of tissues).
