# Supplementary material for: Cosmopolitan Distribution of Endozoicomonas-Like Organisms and Other Intracellular Microcolonies of Bacteria Causing Infection in Marine Mollusks
Source: Front Microbiol. 2020 Oct 30;11:577481. doi: 10.3389/fmicb.2020.577481 (PMC7661492; doi:10.3389/fmicb.2020.577481)

**Supplement S2.** Specific PCR for the detection of *Endozoicomonas*-like organisms (ELO) using the set of primers IMC-F and IMC-R described in Cano et al., 2018. Agarose gels showing a fragment of either 407 bp (first round of PCR, figure B) or 282 bp (second round of PCR, figures A and C) of the 16S rRNA gene. A) wedge clam *Donax trunculus* (samples ID from 1 to 5) and smooth clam *Callista chione* (samples ID from 6 to 9) collected in Italy; B) king scallop *Pecten maximus* collected in France (samples ID 9 to 15); and C) common cockle *Cerastoderma edule* collected in the United Kingdom (samples ID 16 to 19). DNA was extracted either from formalin-fixed paraffin-embedded tissues in duplicate (A) or from ethanol fixed tissues (B and C). Positive DNA of king scallop *Pecten maximus* gill tissue was used as positive control (+ve). Water was used as negative control (-ve). 650 ng of a 100 bp DNA ladder (Promega) was used as molecular weight marker (M).

Samples ID: 1: 16002-1; 2: 16002-2; 3: 16002-3; 4: 16002-4; 5: 16002-5; 6: 16021-1; 7: 16021-2; 8: 16021-3; 9: 16021-4; 10: 09-065-12 gills; 11: 09-65-25 gills; 12: 09-114-02 gills; 13: 09-114-01 gills; 14: 09-114-6 digestive gland; 15: 09-065-01 gills; 16: PM30170-1 gills; 17: PM30170-2 gills; 18: PM30170-3 gills; 19: PM30170-4 gills. Metadata associated can be found in the supplemental table S1.

**
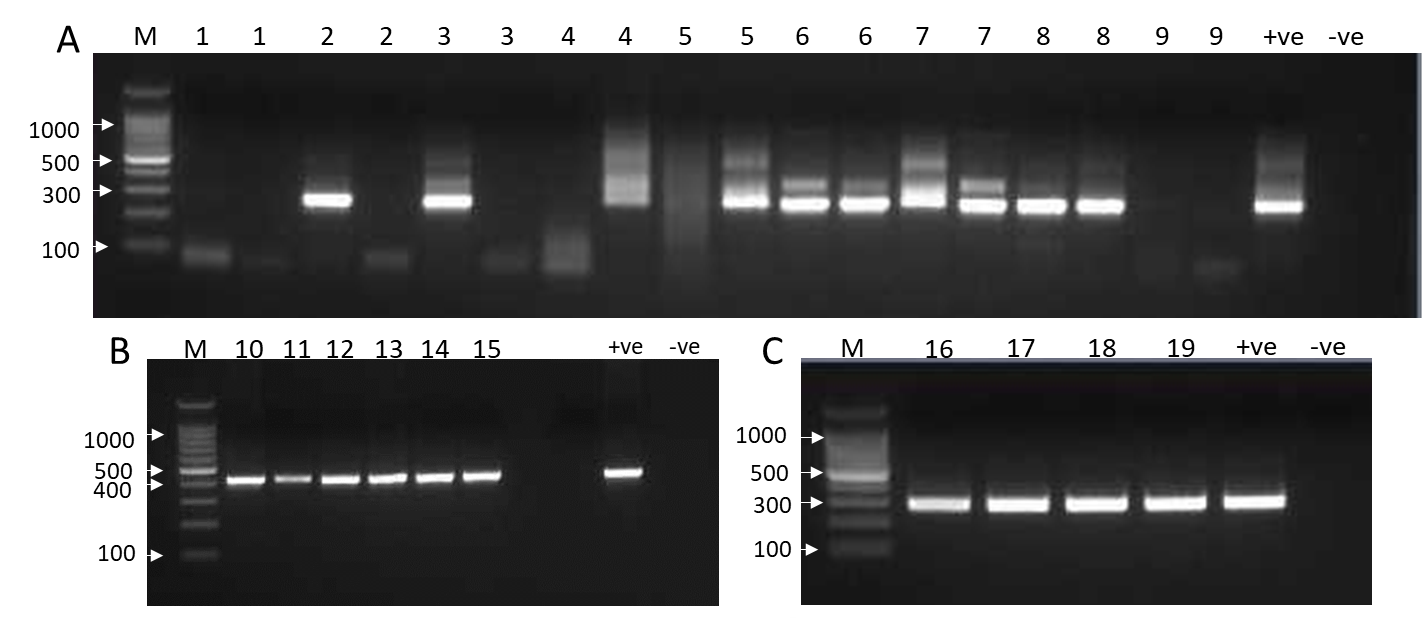
**

**Supplement S3.** Maximum-likelihood tree showing phylogenetic relationships among the 16S rRNA gene of PCR sequenced products and a selection of *Endozoicomonas*-like organisms (ELO) and other symbiotic bacteria*.* Sequence shows the name of the host, sample ID, country of origin, and tissue (G: gill, DG: digestive gland, V: variety of tissues).


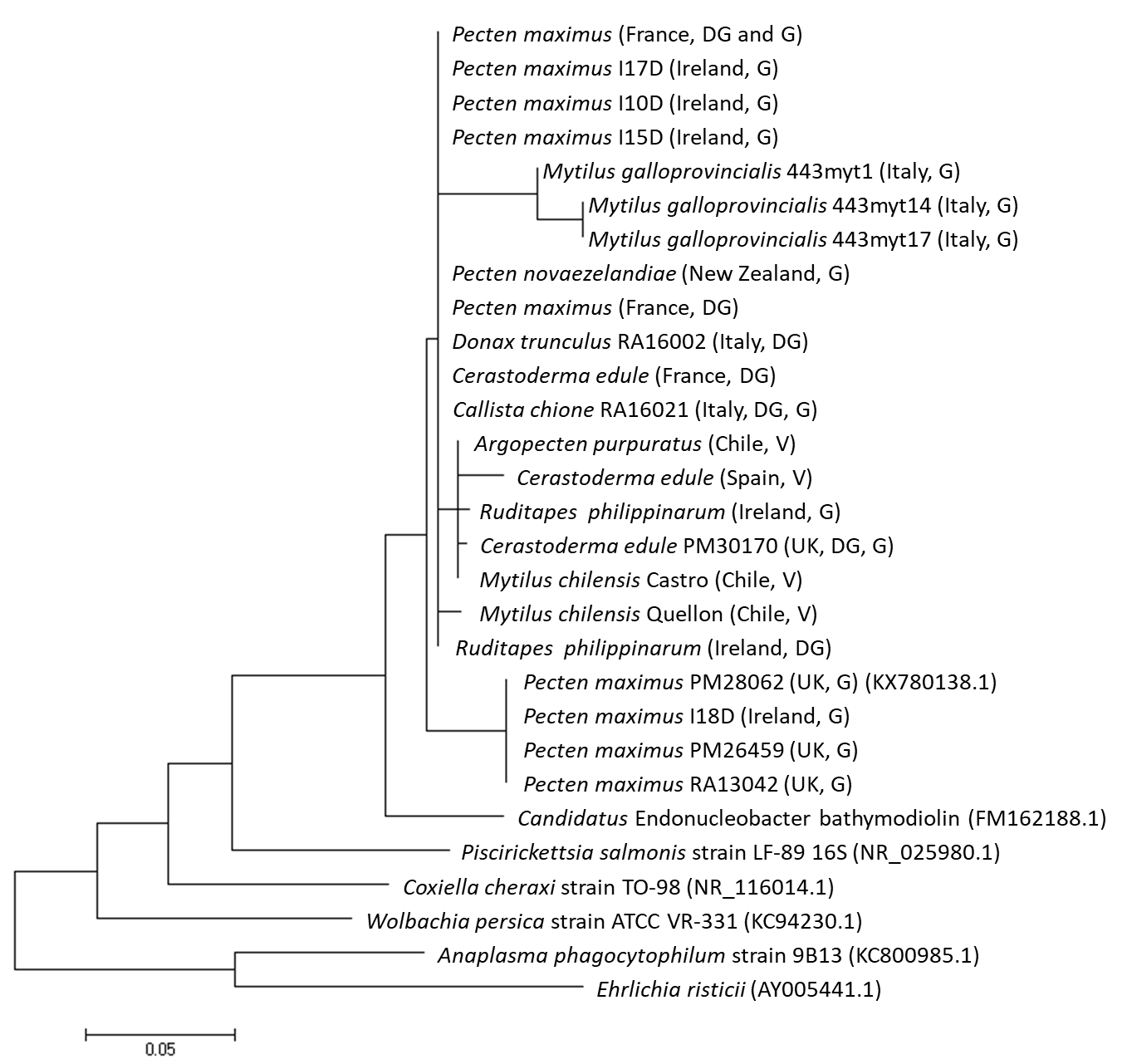

Supplement: Supplementary 2 — Specific PCR for the detection of Endozoicomonas-like organisms (ELO) using the set of primers IMC-F and IMC-R described in Cano et al. (2018). Agarose gels showing a fragment of either 407 bp (first round of PCR, panel B) or 282 bp (second round of PCR, panels A,C) of the 16S rRNA gene. (A) Wedge clam Donax trunculus (samples ID from 1 to 5) and smooth clam Callista chione (samples ID from 6 to 9) collected in Italy. (B) King scallop Pecten maximus collected in France (samples ID 9 to 15). (C) Common cockle Cerastoderma edule collected in the United Kingdom (samples ID 16 to 19). DNA was extracted either from formalin-fixed paraffin-embedded tissues in duplicate (A) or from ethanol fixed tissues (B,C). Positive DNA of king scallop Pecten maximus gill tissue was used as positive control (+ve). Water was used as negative control (−ve). 650 ng of a 100 bp DNA ladder (Promega) was used as molecular weight marker (M). [file Data_Sheet_1.docx]
